# Predictive water virology using regularized regression analyses for projecting virus inactivation efficiency in ozone disinfection

**DOI:** 10.1016/j.wroa.2021.100093

**Published:** 2021-02-12

**Authors:** Syun-suke Kadoya, Osamu Nishimura, Hiroyuki Kato, Daisuke Sano

**Affiliations:** a Department of Civil and Environmental Engineering, Graduate School of Engineering, Tohoku University, Aoba 6-6-06, Aramaki, Aoba-ku, Sendai, Miyagi, 980-8579, Japan; b New Industry Creation Hatchery Center, Tohoku University, Sendai, Miyagi, Japan; c Department of Frontier Sciences for Advanced Environment, Graduate School of Environmental Studies, Tohoku University, Aoba 6-6-06, Aramaki, Aoba-ku, Sendai, Miyagi, 980-8579, Japan

**Keywords:** Log reduction value, Ozone disinfection, Waterborne viruses, Regularized regression analyses, Hierarchical bayesian modeling

## Abstract

Wastewater reclamation and reuse have been practically applied to water-stressed regions, but waterborne pathogens remaining in insufficiently treated wastewater are of concern. Sanitation Safety Planning adopts the hazard analysis and critical control point (HACCP) approach to manage human health risks upon exposure to reclaimed wastewater. HACCP requires a predetermined reference value (critical limit: CL) at critical control points (CCPs), in which specific parameters are monitored and recorded in real time. A disinfection reactor of a wastewater treatment plant (WWTP) is regarded as a CCP, and one of the CCP parameters is the disinfection intensity (*e.g.*, initial disinfectant concentration and contact time), which is proportional to the log reduction value (LRV) of waterborne pathogens. However, the achievable LRVs are not always stable because the disinfection intensity is affected by water quality parameters, which vary among WWTPs. In this study, we established models for projecting virus LRVs using ozone, in which water quality and operational parameters were used as explanatory variables. For the model construction, we used five machine learning algorithms and found that automatic relevance determination with interaction terms resulted in better prediction performances for norovirus and rotavirus LRVs. Poliovirus and coxsackievirus LRVs were predicted well by a Bayesian ridge with interaction terms and lasso with quadratic terms, respectively. The established models were relatively robust to predict LRV using new datasets that were out of the range of the training data used here, but it is important to collect LRV datasets further to make the models more predictable and flexible for newly obtained datasets. The modeling framework proposed here can help WWTP operators and risk assessors determine the appropriate CL to protect human health in wastewater reclamation and reuse.

## Highlights

• The ozone inactivation models to predict virus LRVs are established using regularized regressions.
• The models are applicable to the variety of water quality among wastewater treatment plants.
• The models are relatively strong for extrapolation.
• To develop more robust models, inactivation data with water quality information need to be accumulated.

## Graphical abstract

**Figure:**
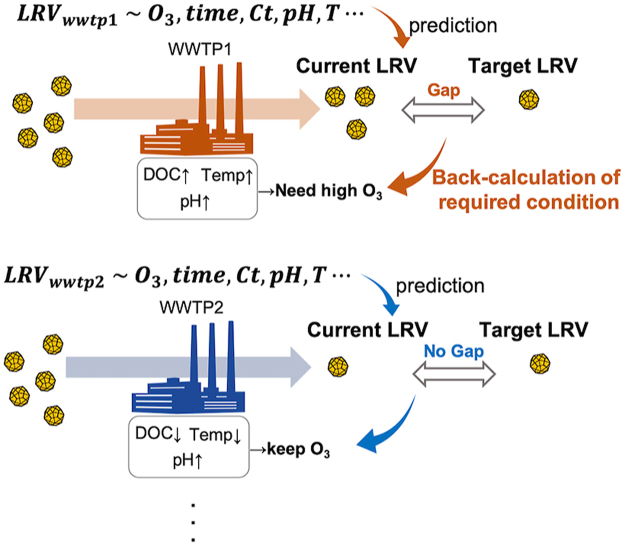

## 1 Introduction

Water shortages are a critical global concern and endanger human lives all over the world. Wastewater reclamation and reuse have significantly contributed to the improvement of the accessibility of safe water, but waterborne viruses such as norovirus, rotavirus, adenovirus and enterovirus have been detected from effluents of wastewater treatment plants (WWTPs) (Subahir et al., 2019; Hoque et al., 2018; Croker et al., 2018; Bauri et al., 2019; Li et al., 2018; Silva-Sales et al., 2020; Katayama et al., 2008; Adeifisove et al., 2016; Shlindwein et al., 2010). The risk of viral infection to humans, including users of reclaimed wastewater, needs to be addressed because waterborne viruses in wastewater have imposed a significant disease burden on human society (Utsumi et al., 2020; Subahir et al., 2019; Hoque et al., 2018; Croker et al., 2018; Bauri et al., 2019; Li et al., 2018).

International and domestic guidelines for wastewater reclamation and reuse employ the multiple-barrier concept to address the waterborne disease risk in the usage of reclaimed wastewater, in which the target log reduction value (LRV) of waterborne pathogens is determined beforehand, and the target LRV is allocated to each step at a wastewater treatment plant (WWTP) (Ito et al., 2017; Sano et al., 2016). The World Health Organization (WHO) has recommended applying Sanitation Safety Planning (SSP) to the safe use of excreta, wastewater and greywater, and the hazard analysis and critical control point (HACCP) approach is employed in SSP in order to manage human health risks upon exposure to reclaimed wastewater (WHO, 2006 and 2015). The HACCP approach includes the identification of critical control points (CCPs), which are important operational steps to determine the magnitude of hazardous factors in the final products (U.S. FDA, 1997). The parameters at the CCP are monitored and recorded in real time. In addition, the parameters are compared to reference values called critical limit (CL) to maintain the safety of final products (U.S. FDA, 1997). The corrective action needs to take place when the parameters monitored at CCP deviate from CL. The disinfection intensity, determined by some operational parameters such as Ct-value in a disinfection reactor, is suitable as a CCP parameter at WWTPs since it determines the LRV of waterborne pathogens at a disinfection reactor.

However, an application of SSP to the WWTP operation is difficult at present since a universal method for CL determination has not yet been developed. In addition, the difference in water quality among WWTPs makes it difficult to establish a universal approach that determines the CLs appropriate for respective WWTPs, because chemical disinfectants are consumed by contaminants in wastewater, and the decay of disinfectant concentration results in deviation from the expected disinfection intensity. Thus, the disinfection intensity that achieves the target LRV needs to be determined by taking water quality and operational information at each WWTP into account. Previously, we proposed the concept of predictive water virology, in which the models predicted virus LRV by using water quality and operational parameters as explanatory variables (Kadoya et al., 2019). The predictive water virology enables us to derive the appropriate CL to attain the target LRV under site-specific water quality. Kadoya et al. (2019 and 2020) previously proposed the concept of predictive water virology and reported that the predictive inactivation models, based on hierarchical Bayesian modeling (HBM) and regularized regressions, flexibly correspond to the changes and variety in water quality parameters among WWTPs (*e.g.,* seasonal variation). Predictive water virology can help us understand the required operational conditions to achieve the target LRV, in which there is no gap between the predicted and target LRVs. However, these previous studies were limited in their predictions of newly obtained datasets that are not used for model construction, in other words, we need to know the predictable range of present models and identify which algorithms are more robust to predict LRVs using new datasets.

Basic regularized regression analyses are ridge, lasso and elastic net regressions, in which the multicollinearity problem is solved, and thus all explanatory variables can be used. All these regression approaches are extended versions of the least square method by adding a regularization term corresponding to each algorithm. The ridge regression shrinks absolute values of the coefficients of model variables (Hoerl and Kennard, 1970), whereas the lasso regression makes the coefficients of inessential variables zero with a decrease in the magnitude of model variables (Tibshirani, 1996). The elastic net regression combines regularized terms used in both the ridge and lasso regressions (Zou and Hastie, 2006). The lasso and elastic net regressions are classified into the sparse estimation, which selects the plausibly essential variables. In Bayesian regularized regression (Bayesian ridge and automatic relevance determination (ARD)), an objective variable is assumed to be expressed as Gaussian functions, and regularization terms are then estimated as hyperparameters by Bayes rule. A Bayesian ridge employs all explanatory variables as well as basic ridge regression, whereas ARD is also one of the sparse estimations (Tipping, 2001). Previous studies have applied several machine learning algorithms to establish predictive models for water quality and rainfall runoff (Park et al., 2018; Lu and Ma, 2020; Safari et al., 2020), but the predictive inactivation models for waterborne viruses, which are based on Bayesian regularized regression analyses using water quality and operational parameters, have not been reported.

In this study, we introduced the Bayesian regularized regression analysis into the predictive water virology to explore appropriate algorithms, which indicated the best prediction performance and were capable of predicting newly obtained datasets and avoiding overfitting to training datasets. We here have focused on inactivation datasets related to ozone, which is one of disinfectants effective for inactivation of waterborne viruses (Roy et al., 1981; Kim et al., 1980) and is utilized at many WWTPs (Hembach et al., 2019; Kharel et al., 2020; Wang et al., 2018). We first collected peer-reviewed articles following a systematic review method and extracted LRV datasets with water quality and operational information from water disinfection experiments using ozone. We applied three regularized regressions (ridge, lasso, and elastic net) and two Bayesian regularized regressions (Bayesian ridge and ARD) to construct the predictive inactivation models, and then verified the prediction performance and overfitting problem based on two validation approaches: (1) the models constructed by training datasets (80% of total dataset) predicted test datasets (20% of total dataset) for evaluation of the prediction performance, and (2) one article was selected and its dataset was used as a test dataset, which had no relation to the training datasets, to confirm whether the model overfitted. We then tried to combine the best regularized regression-based models with HBM, which took experimental errors and virus type-dependent sensitivity into account (Kadoya et al., 2019). Finally, we provided the applicable range of explanatory variables to help readers obtain more precise prediction values and the required disinfection conditions under the water quality level to achieve 4 LRV, which has been recommended by the USEPA (USEPA, 1999).

## 2 Methods

### 2.1 Systematic Review and data collection

Peer-reviewed articles relating to virus inactivation using ozone were collected using Google Scholar from April 2019 to June 2020, following PRISMA guidelines (Moher et al., 2009). The keywords input to Google Scholar were “disinfection” and “ozone” combined with keywords of each virus species (“norovirus”, “rotavirus”, “poliovirus”, “adenovirus”, “hepatitis A virus”, “coxsackievirus” and “echovirus”), and we checked all records published from 1950 to 2020. Dissertations, book chapters, reviews, conference reports and non-English articles were eliminated from the collected articles (first screening). We read whole texts in the first-screened records, and the articles relevant to this study aim, which included the information about the virus LRV and ozone disinfection conditions such as contact time, initial concentration and Ct-value, were then selected from the first-screened records. To establish models using the highly qualified datasets, we assumed that LRVs must be differentiated under varied conditions of disinfection in well-performed experiments, in which water quality and operational parameters were strictly controlled. If the LRVs were same in spite of distinct disinfection conditions, the datasets were excluded. The process of systematic review was fulfilled by three persons.

The LRV was expressed as below:

(1) $$LRV=log_{10}\left(N_{0}/N_{t}\right)$$

where *N*_*0*_ is virus concentration at time 0 and *N*_*t*_ is that at time *t*. ImageJ software was used to extract numerical datasets of the LRV from figures, such as an inactivation curve (Shneider et al., 2012). Additionally, we obtained water quality and operational parameters such as initial ozone concentration (*I*) [mg/L], decay rate of ozone concentration (*k*) [min^-1^], contact time (*t*) [min], Ct-value (*C*) [min mg/L], pH (*p*) [-], temperature (*T*) [°C], assays for measuring virus concentration (*A1*: qPCR or infectivity, *A2*: plaque assay or MPN method when *A1* is infectivity), types of water used for the disinfection experiment (*W*: purified or environmental water) and log_10_ initial virus concentration (*V* [log_10_ (genome copy number, PFU or MPN/mL)]). Virus types (*S*) were also extracted from the collected articles (Table S1), of which rotavirus and coxsackievirus were used as categorical variables because they are the only two types that were tested in the articles. When datasets did not include Ct-values, we calculated them by an integration of the disinfectant decay formula (Eq. (2)):

(2) C(t)=Iexp(−kt)

where *C(t)* is ozone concentration at time *t* [min], *I* is initial concentration of ozone and *k* is the decay constant of ozone concentration in water.

### 2.2 Basic regularized regression analysis

Explanatory variables in the regularized regression analyses were *I*, *k*, *t*, *C*, *p*, *T*, *A1*, *A2*, *W*, *V* and *S,* indexed above. The variables *A1*, *A2*, *W* and *S* were binary ones (0: genome copy (in *A1*), PFU (in *A2*), purified water and one virus type, 1: infectivity (in *A1*), MPN (in *A2*), environmental water, another type). Interaction and interaction-quadratic terms were added in a stepwise procedure using the “PolynomialFeatures” function of the scikit-learn library (Pedregosa et al., 2011) in Python 3.7. Regularized regression analysis requires a standardization of explanatory variables (mean = 0 and standard deviation = 1), which was performed by the function “StandardScaler” in scikit-learn library.

The response variable LRVs (***y***) were expressed as a linear function:

(3) ***y*** = ***Xβ*** + ***ε***

where ***y*** is a matrix of response variables of (*y*_*1*_, …, *y*_*ρ*_), ***β*** is a coefficient matrix (*β*_*1*_*, …, β*_*ρ*_), ***ε*** is the matrix of observation errors of (*ε*_*1*_,..., *ε*_*ρ*_), ***X*** is the design matrix of model variables of (*x*_*(1)*_, ..., *x*_*(ρ)*_), *x*_*(j)*_ is a matrix of (*x*_*1j*_, ..., *x*_*nj*_), *ρ* is the number of datasets, and *n* is the number of model variables). Minimizing the sum of squares error (*S*_*λ*_) derives the estimates of coefficient:

(4) $$min_{\beta }S_{\lambda }=min_{\beta }\left\{\frac{1}{2n}{\left\|y-X\beta \right\|}_{2}^{2}+\lambda R\left(\beta \right)\right\}$$

where *min*_***β***_ is a minimization function for ***β***, *λ* is the regularization parameter and a regularization term R(***β***) determines which regularization methods are used for constructing models. R(***β***) for ridge regression is expressed as $1/2{\left\|\beta \right\|}_{2}^{2}$ (${\left\|\beta \right\|}_{2}^{2}=\overset{n}{\underset{i=1}{\sum }}\beta_{i}^{2}$) (Hoerl and Kennard, 1970) while for lasso regression $\left\|\beta_{1}\right\|$ ($\left\|\beta_{1}\right\|=\overset{n}{\underset{i=1}{\sum }}\left|\beta_{i}\right|$) is applied (Tibshirani, 1996). Elastic net regression combines the R(***β***) of ridge and lasso regressions ($\alpha {\left\|\beta \right\|}_{2}^{2}+\left(1-\alpha \right){\left\|\beta \right\|}_{1}$, where *α* determines the proportion of the ridge to the lasso regression) (Zou and Hastie, 2006). Suitable values of two parameters (*λ* and *α*) were concurrently searched from 10^-6^ to 10^2^ and from 0 to 1, respectively, using the grid search method that enumerated all combinations of those parameters and identify an appropriate combination. The standardization of variables and the addition of regularization term can ensure the homoscedasticity of errors.

### 2.3 Bayesian regularized regression analysis

Regularization parameters are determined not by fitting to data but by the random procedure in the grid search method as described above. Bayesian regularized regression analysis enables us to estimate regularization parameters with coefficients of regression models by fitting to data, and can avoid overfitting by making the model less complex (Tipping, 2001).

The coefficient ***β*** follows a spherical Gaussian distribution in Bayesian ridge regression analysis, and two regularization parameters (***f*** and *σ*^*2*^) are given by gamma distributions as prior distributions (Tipping, 2001; Bishop, 2006). In this case, the likelihood of ***y*** is expressed as:

(5) $$p\left(y|\beta ,\;\sigma^{2}\right)={\left(2\pi \sigma^{2}\right)}^{-\rho /2}exp\left\{-\frac{1}{2\sigma^{2}}{\left\|y-\beta X\right\|}^{2}\right\}$$

The prior for ***β*** is described as:

(6) $$p\left(\beta |f)=\overset{\rho }{\underset{i=0}{\prod }}N(\beta_{i}|0,\;{f_{i}}^{-1}\right)$$

and the prior distributions for ***f*** and *σ*^*2*^ are:

(7) $$p\left(f\right)=\overset{\rho }{\underset{i=0}{\prod }}Gamma\left(f_{i}|a,\;b\right)$$

(8) $$p\left(\sigma^{2}\right)=Gamma\left(\sigma^{2}|c,\;d\right)$$

where *a* and *c* are shape parameters, and *b* and *d* are rate parameters of each gamma distribution. These parameters (*a*, *b*, *c* and *d*) were determined from non-informative distributions. Maximizing the logarithmic posterior distribution (ln *p*(***β***|***y***)) is equal to the minimization of a regularization term. Automatic relevance determination (ARD) employs the elliptical Gaussian distribution for a prior distribution of ***β***, and follows the same procedure of Bayesian ridge regression (Tipping, 2001; Bishop, 2006). These Bayesian regularized regression analyses were also conducted by the scikit-learn library in Python 3.7; codes and source data are available in the supplementary materials.

### 2.4 Hierarchical bayesian modeling

We adopted probability distributions specific to the objective variables’ LRV based on Akaike’s information criterion (AIC) using the “fitdistrplus” packages of R software (Delignette-Muller and Dutang, 2015). The population parameter shaping the probability distribution (***Y***) was described as a logarithmic link function because the LRV datasets followed a Weibull or gamma distribution (Table S2). The logarithmic link function is expressed as below:

(9) $$log_{e}Y=\beta_{0}+\beta_{E}X$$

where *β*_*0*_ is an intercept, ***β***_***E***_ is a matrix of coefficients and ***X*** is a design matrix. We assumed that LRVs involved an experimental error, and the matrix of coefficients was indexed “***E***”. The ***β***_***E***_ was assumed to follow a normal distribution because of experimental error:

(10) $$\beta_{E}∼Normal\left(\mu_{\mathrm{ST}\left[i\right]},\;\sigma_{E}\right)$$

where *σ*_*E*_ is a standard deviation generating variations among inactivation experiments, and ***μ***_***ST[i]***_ is a mean of virus type *i*, which implies the type-dependent sensitivity to ozone. The virus type-dependent parameter ***μ***_***ST[i]***_ also follows a normal distribution:

(11) $$\mu_{\mathrm{ST}\left[i\right]}∼Normal\left(\mu_{O},\;\sigma_{ST}\right)$$

where ***μ***_***O***_ is the averaged mean among all virus types and *σ*_*ST*_ is the standard deviation bearing the type-dependent sensitivity. The hierarchical Bayesian models were constructed by two approaches that employed explanatory variables selected by the best regularized regression models or variance inflation factor (VIF). A multicollinearity problem possibly remained in the HBM, so the models that removed an explanatory variable having more than 10 VIF values were prepared. HBM was conducted on R software version 3.5.0 (https://www.r-project.org/) and Stan (https://mc-stan.org/).

### 2.5 Model validation

We conducted two trials to determine which regularized regression algorithms exhibited the best prediction performance and escaped overfitting to training datasets. In Trial 1, we randomly separated total datasets into training (80%) and test datasets (20%) to evaluate the prediction powers for interpolation. In Trial 2, the datasets derived from an article were used as test datasets, which resulted in no relationship between training and test datasets and enabled us to understand the predictability for extrapolation. Both trials adopted leave-one-out (LOO) cross-validation, in which temporal models were repeatedly generated using *N-1* training datasets (*N* is the number of training datasets), and a remaining dataset was used for model validations. The coefficients were the averaged values of the generated *N* models. To evaluate the prediction performance, we calculated mean squared values (MSEs). We also generated a number of the predicted LRVs by changing, stepwise, some explanatory variables having a higher coefficient to explore the out-of-predictable range in which a model generated obviously wrong prediction values such as a negative LRV.

## 3 Results

### 3.1 Article collection and data characteristics

We first identified 10102 records according to a systematic review method. Dissertations, book chapters, reviews and non-English articles were eliminated from the list of the first-collected articles, which resulted in a decrease to 4058 articles. After excluding the articles not relevant to our study (*e.g*., no information about the LRV and no water disinfection), 21 articles remained. In detail, the numbers of screened articles for norovirus, rotavirus, poliovirus, coxsackievirus, echovirus and adenovirus were 6, 4, 12, 4, 3 and 2, respectively. The numbers of LRV data point were 68 (norovirus), 65 (rotavirus), 182 (poliovirus), 111 (coxsackievirus), 29 (echovirus) and 29 (adenovirus), respectively (Lim et al., 2010; Brie et al., 2018; Hirneisen et al., 2011; Tondera et al., 2015; Shin and Sobsey, 2003; Thurston-Enriquez et al., 2005; Harakeh and Bulter, 1984a; Harakeh and Bulter, 1984b; Meunier et al., 2006; Vaughn et al., 1987; Finch and Fairbairin, 1991; Farooq and Akhlaque, 1983; Katzenelson and Biedermann, 1976; Moore and Magolin, 1994; Roy et al., 1981; Roy et al., 1982; Engelbrecht et al., 1980; Katzenelson et al., 1979; Emerson et al., 1982; Wang et al., 2018; Wolf et al., 2018). The Scikit-learn library (https://scikit-learn.org/stable/tutorial/machine_learning_map/index.html) and previous studies suggest that more than 50 datasets should be analyzed (Cui and Gong, 2018; Leonard et al., 2017), so we excluded echovirus and adenovirus data from our analyses.

**Fig. 1:**
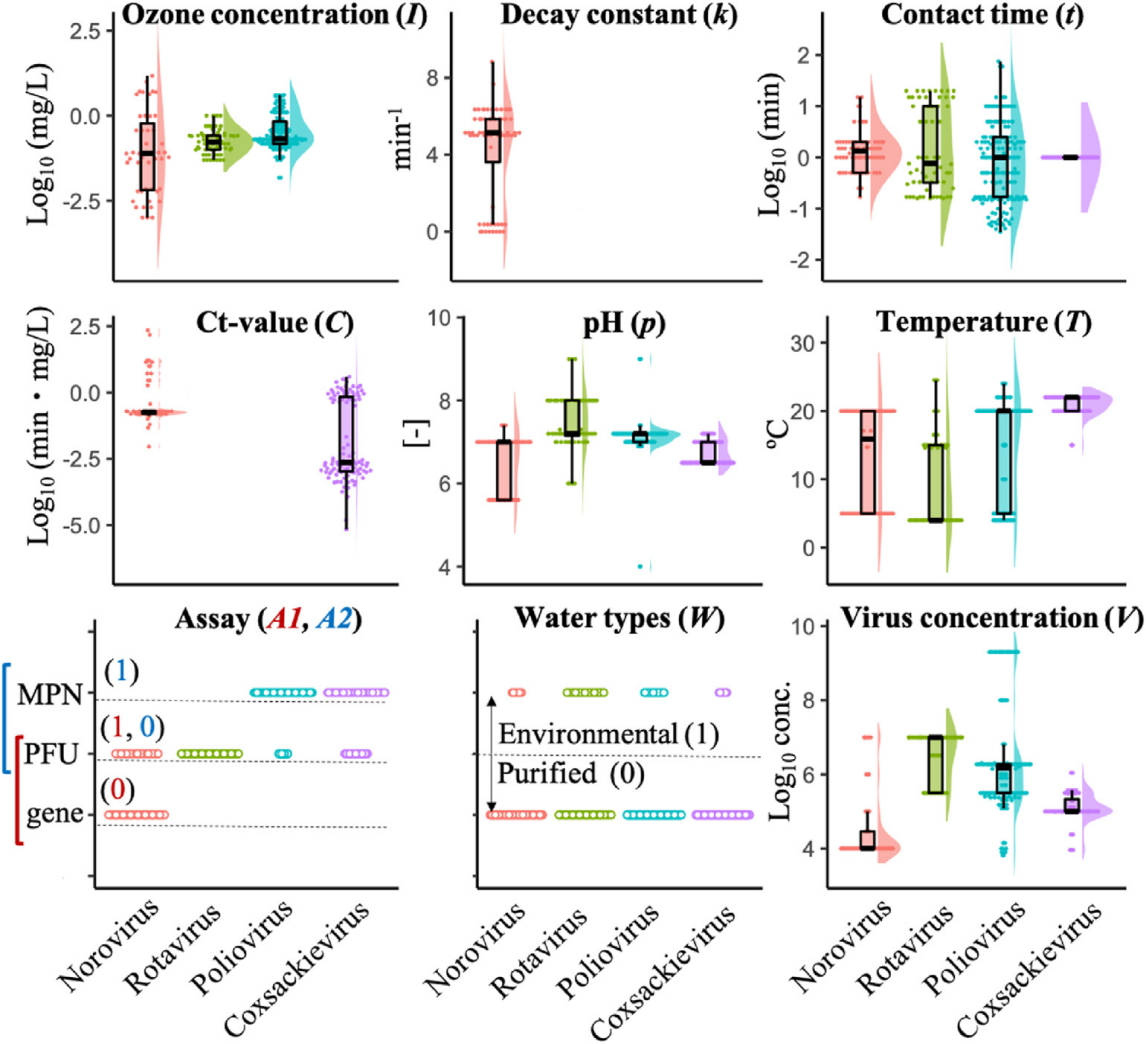
**Distribution of operational and water quality parameters**. Water quality and operational parameters except for strain types were plotted for each virus species as swarm, box (median, 25 and 75 percentile) and violin plots. “Assay” is classified as A1 (genome or infectivity) or A2. (If classifying as infectivity, the variable is also classified as plaque forming unit (PFU) or most probable number (MPN) method.).

Water quality and operational parameters were also extracted from the screened articles. Initial concentrations of ozone (*I*) are mostly less than 5 mg/L (Fig. 1, *I*), and the decay constant (*k*) is described only in articles for norovirus (Fig. 1, *k*). Data points of the contact time (*t*) gather around lower values, but a part of the data for rotavirus and poliovirus was distributed around 10 min (Fig. 1, *t*). Ct-values (*C*) were not listed in articles about rotavirus and poliovirus (Fig. 1, *C*). The articles about coxsackievirus listed *C* but included no descriptions of *I*. The values of pH (*p*) are centered around 7 ± 1 (Fig. 1, *p*), and almost all disinfection experiments are conducted at room temperature (around 20 °C) or 4 °C (Fig. 1, *T*). Purified water, like a buffer, was mainly used for disinfection tests (Fig. 1, *W*). Infectivity assay (plaque assay or MPN method) is one of the main methods to measure virus concentration (Fig. 1, *A1* and *A2*). Initial log virus concentration ranges from approximately four to eight (Fig. 1, *V*). Since the collected studies used only two types of rotavirus and coxsackievirus (Table 1 and Table S1), the types were regarded as an explanatory variable in the regularized regression analyses. Taken together, the number of explanatory variables was 9 (norovirus), 8 (rotavirus), 7 (poliovirus) and 7 (coxsackievirus) (Table 1).

**Table 1:** Information about datasets used for the model construction (*N*_*LRV*_: the number of log reduction values, *N*_*E*_: the number of articles, *N*_*S*_: the number of virus types, *I*: initial ozone concentration, *k*: decay constant, *C*: Ct-value, *p*: pH, *T*: temperature, *A1*: infectivity or genome, *A2*: plaque forming unit or most probable number method, *W*: water types, *V*: log_10_ initial virus concentration).

	*N*_*LRV*_	*N*_*E*_	*N*_*S*_	Parameter	Ref
Norovirus	68	6	4	*I, k, t, C, p, T, A1, W, V*	Lim et al. (2010)
					Brie et al. (2018)
					Hirneisen et al. (2011)
					Tondera et al. (2015)
					Shin and Sobsey (2003)
					Thurston-Enriquez et al. (2005)
Rotavirus	65	4	2	*I, t, p, T, A1, W, V, S*	Harakeh and Bulter (1984a)
					Harakeh and Bulter (1984b)
					Meunier et al. (2006)
					Vaughn et al. (1987)
Poliovirus	182	12	4∗	*I, t, p, T, A1, A2, W, V*	Shin and Sobsey (2003)
					Harakeh and Bulter (1984a)
					Harakeh and Bulter (1984b)
					Finch and Fairbairin (1991)
					Farooq and Akhlaque (1983)
					Katzenelson and Biedermann (1976)
					Moore and Magolin, 1994
					Roy et al. (1981)
					Roy et al. (1982)
					Engelbrecht et al. (1980)
					Katzenelson et al. (1979)
					Emerson et al. (1982)
Coxsackievirus	111	4	2	*C, p, T, A2, W, V, S*	Harakeh and Bulter (1984a)
					Emerson et al. (1982)
					Wolf et al. (2018)
					Engelbrecht et al. (1980)

### 3.2 The prediction performance of regularized regression models in trial 1

**Fig. 2:**
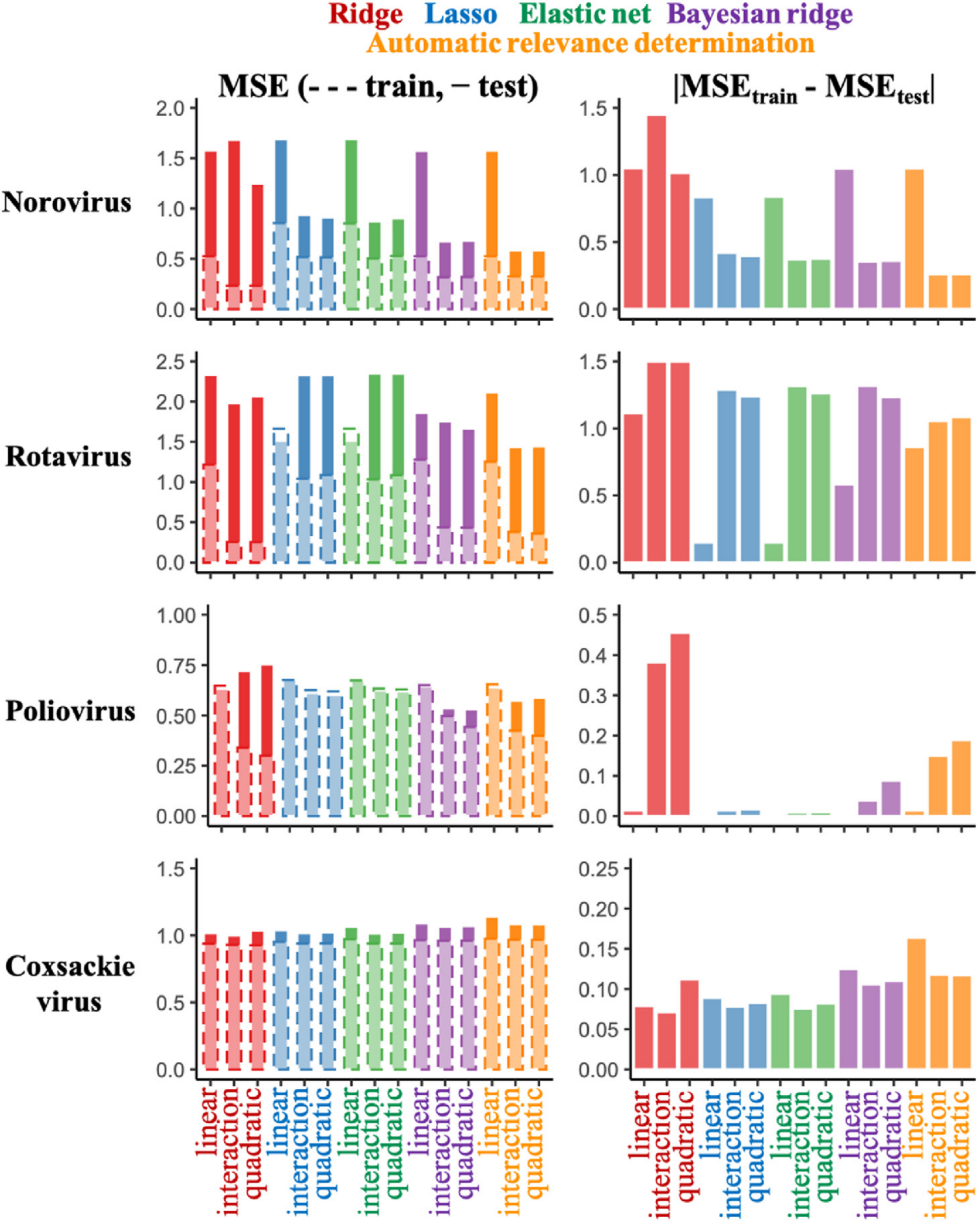
**Mean squared errors (MSEs) in Trial 1.**Total datasets are randomly divided into test and training datasets in Trial 1. MSEs are compared between test (solid) and training (dashed) datasets (left), and the absolute values of difference between test and training data are also displayed (right).

Based on training datasets randomly split from the total dataset, predictive inactivation models for four virus species were constructed. MSEs for both training and test datasets were calculated to identify the best algorithms for the LRV prediction and the required polynomial terms (Fig. 2). The addition of higher terms to the norovirus inactivation model made prediction performances for both datasets better than only linear terms and decreased the differences of MSEs between training and test data (*Δ*MSE = |MSE_train_ - MSE_test_|) except for ridge regression. Also, MSEs decreased by adding interaction terms to the rotavirus inactivation models, whereas *Δ*MSEs in the models with only linear terms were lower. Among models for predicting poliovirus LRV, the ridge regression with polynomial terms indicated the best prediction performance, and Bayesian regularized regression-based models with higher terms also showed smaller MSEs. The coxsackievirus inactivation models were improved very slightly by the addition of polynomial terms, and the ridge-based model with linear terms had the smallest *Δ*MSE. The algorithms providing better prediction performance were ARD with interaction terms (norovirus and rotavirus) and ridge with interaction terms (poliovirus and coxsackievirus) at this time.

### 3.3 The prediction performance for new datasets (trial 2)

**Fig. 3:**
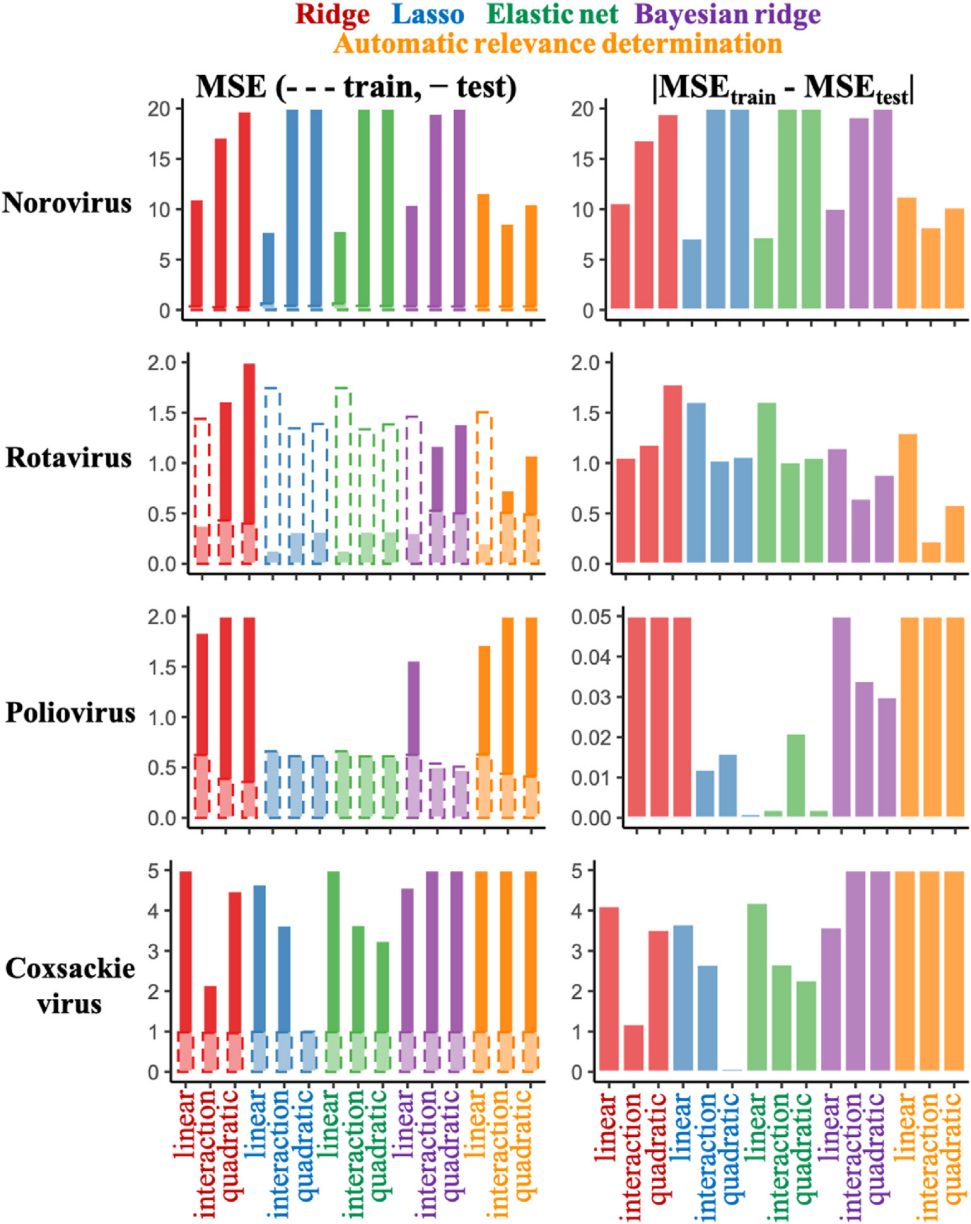
**Mean squared errors (MSEs) in Trial 2.**Datasets from an article are used as test datasets in Trial 2. MSEs are compared between test (solid) and training (dashed) datasets (left), and the absolute values of difference between test and training data are also displayed (right).

In Trial 1, there could be relationships between training and test datasets, which made it difficult to confirm the ability to predict newly obtained datasets. Therefore, we selected datasets extracted from an article as test datasets in Trial 2 to validate the models appropriate for predicting new datasets not related to training datasets. The model indicating smaller MSE and *Δ*MSE is preferable for the predictive inactivation model. In norovirus models, lasso and elastic net with linear terms and ARD with interaction terms indicated smaller MSE and *Δ*MSE (Fig. 3). Only ARD with interaction terms in rotavirus models seemed to avoid the overfitting problem. All poliovirus models had very small *Δ*MSE and smaller MSEs, and Bayesian regularized regressions with polynomial terms slightly improved prediction performances. The coxsackievirus model based on lasso regression with quadratic terms had lower MSEs and *Δ*MSE.

### 3.4 The best predictive inactivation models and their characteristics

**Fig. 4:**
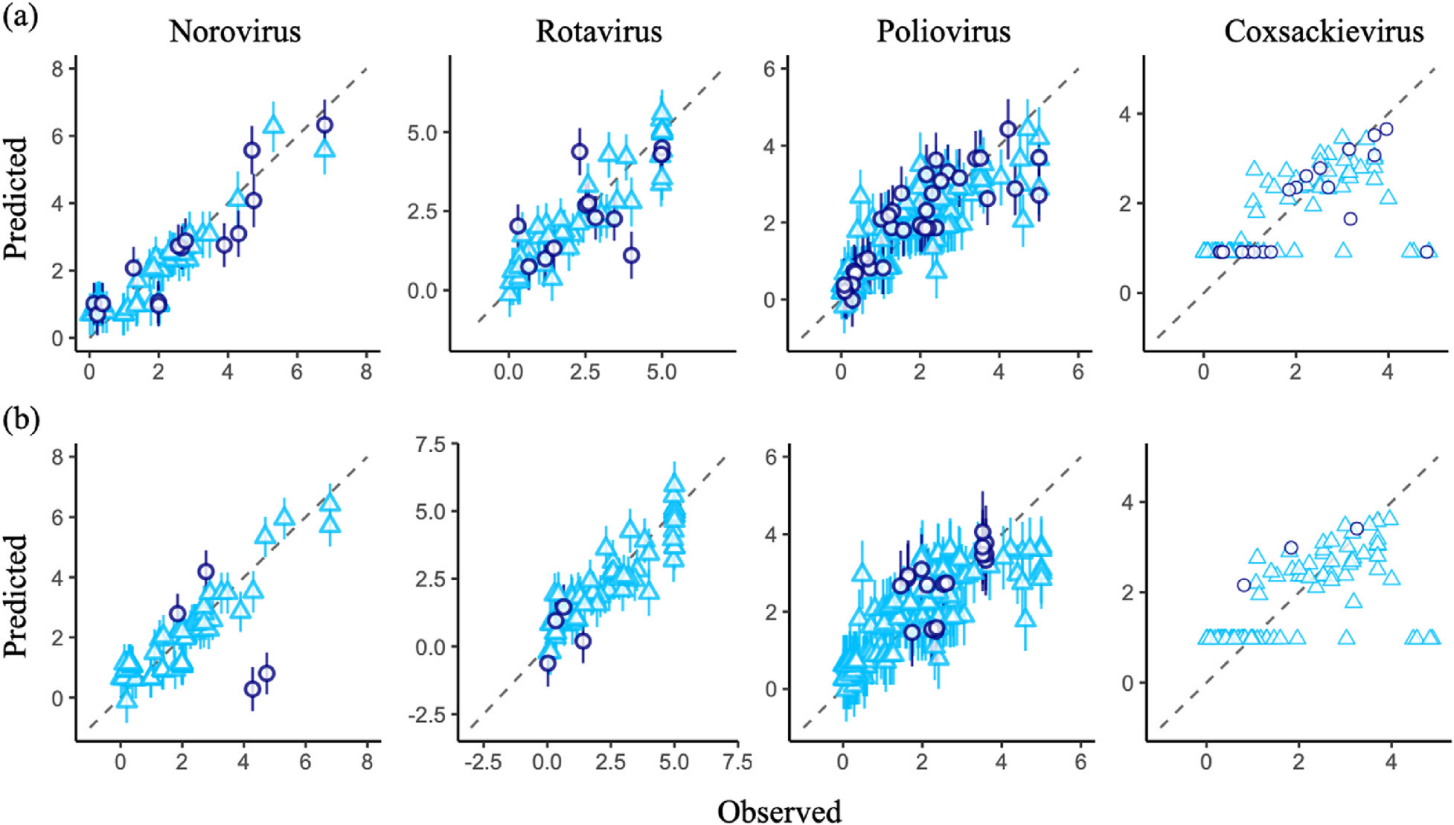
**Comparison of observed data with predicted values by the best predictive models**.Prediction values in the best predictive inactivation models are compared with observed data in the condition of Trial 1 (a) and 2 (b).Triangles and circles are coordinates (observed, predicted) for training and test datasets, respectively. Gray dotted lines indicate a predicted value completely matches an observed point. Error bar means a standard deviation.

**Fig. 5:**
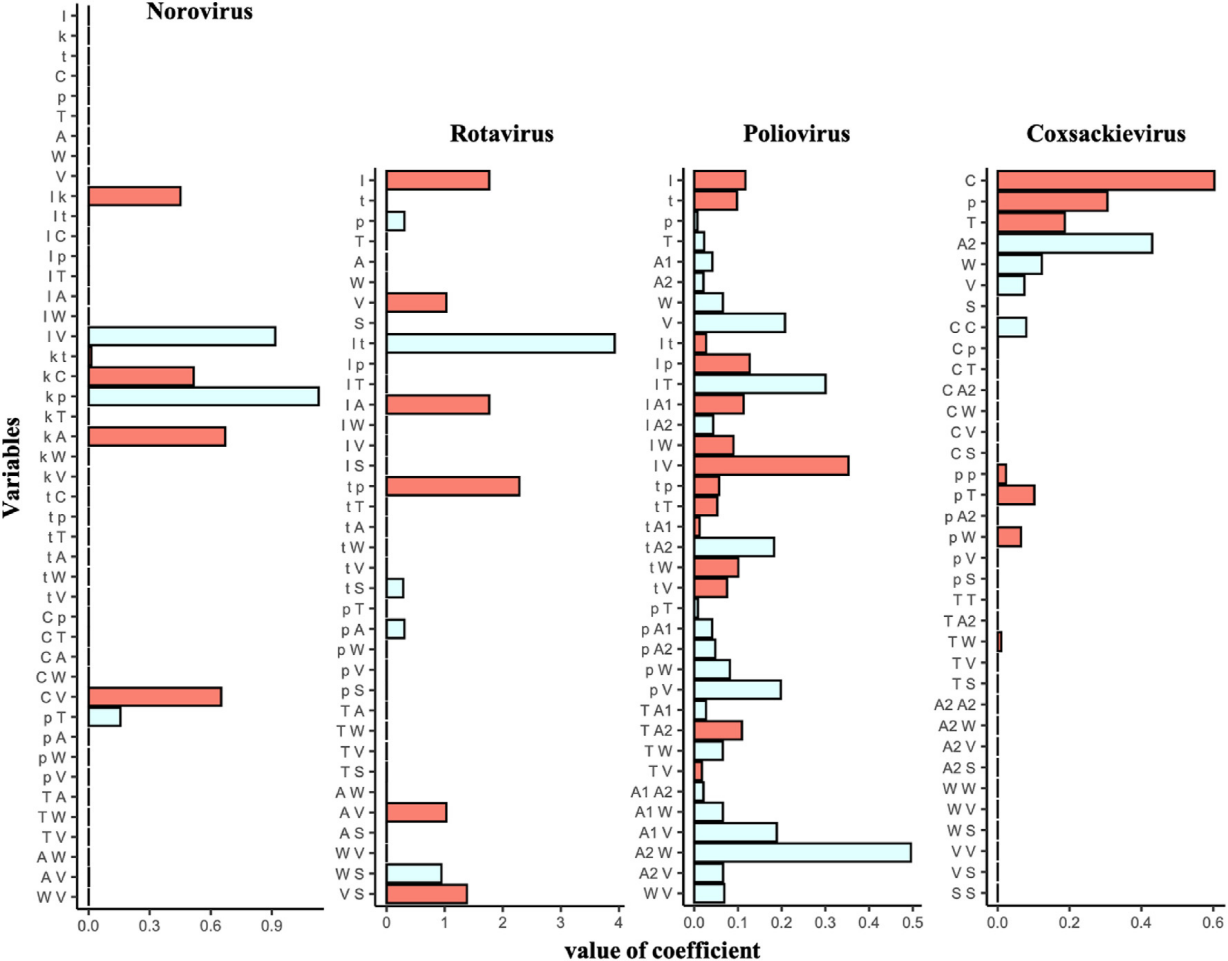
**Model structures for the best predictive inactivation models**. Coefficients of the best models (red: positive, pale blue: negative). Letters indicate explanatory variables (*I*: initial ozone concentration, *k*: decay constant, *t*: contact time, *C*: Ct-value, *A*: assays to measure virus concentration, *W*: water types, *p*: pH, *T*: temperature, *V*: initial virus concentration). (For interpretation of the references to colour in this figure legend, the reader is referred to the Web version of this article.)

Taken together with the results from Trial 1 and 2, the best predictive inactivation models were ARD with interaction terms for norovirus and rotavirus, Bayesian ridge with interaction terms for poliovirus and lasso with quadratic terms for coxsackievirus (Table 3). Comparative results for observed and predicted LRVs indicated that not only training datasets but also many of the test datasets were predicted well by the best models, but some predicted values (two test data of norovirus in Trial 2, both training and test data of coxsackievirus in Trial 1 and 2) largely deviated from the observed values (Fig. 4). Many of prediction values tended to be on the *y* = *x* line and some were randomly deviated from the line (*i.e.*, residuals are distributed randomly), which indicates the independence of residuals. The estimated coefficients of explanatory variables in the best prediction models are displayed in Fig. 5. Except for the poliovirus model, the number of explanatory variables decreased because of the sparse estimations. All models had the variables directly related to the disinfection condition, such as *I*, *C* and *t*, indicating that they are essential to explain LRVs. Other water quality parameters are also included in the best models although some of them are interaction terms.

### 3.5 No improvement of the prediction performance by HBM

We designed VIF-based and regularized-regression-based hierarchical Bayesian models (Table S3) and compared MSE values among them (Table 2). The hierarchical Bayesian models based on regularized regression analyses showed higher prediction performances than those based on VIF. However, the prediction performances in HBM for test datasets of norovirus, poliovirus and coxsackievirus were lower than the best regularized regression models, indicating that the HBM did not contribute to an improvement of the predictive inactivation models using ozone (Figs. S1 and 2).

**Table 2:** Prediction performance of regularized regression and two types of hierarchical Bayesian models (regularized regression and variance inflation factor (VIF)-based).

	The best regularized regression model		Hierarchical Bayesian model (Regularized regression base)		Hierarchical Bayesian model (VIF base)	
	train	test	train	test	train	test
Norovirus	0.35	8.58	0.55	9.51	0.58	17.8
Rotavirus	0.51	0.73	0.67	0.33	1.34	0.31
Poliovirus	0.54	0.51	–	–	0.79	2.24
Coxsackievirus	0.98	1.06	1.16	4.85	1.17	3921

### 3.6 Identification of disinfection conditions achieving 4LRV and unpredictable ranges

**Fig. 6:**
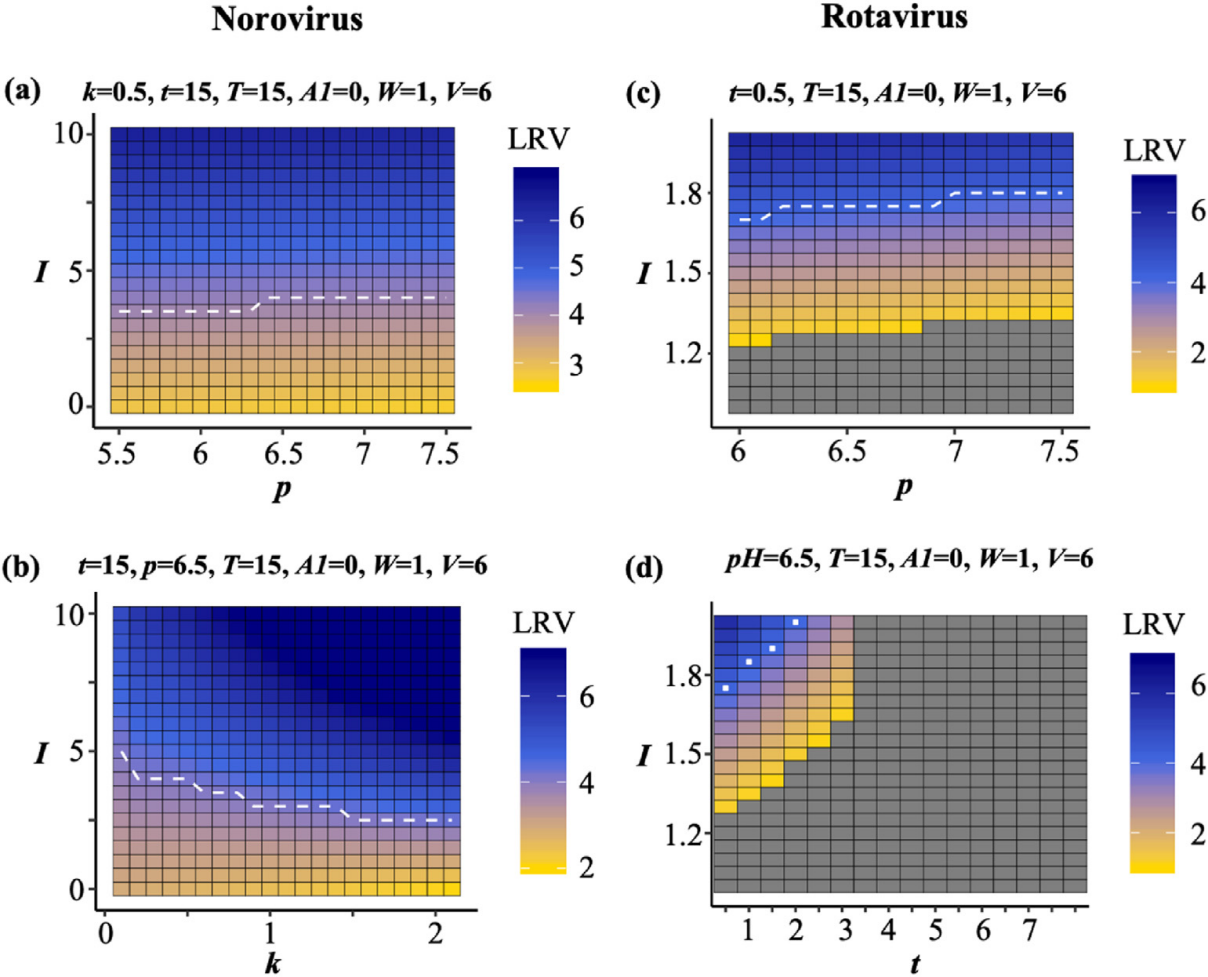
**Simulation of norovirus and rotavirus LRVs**.Heatmaps indicate the LRVs by changing specific explanatory variables, and the white dotted lines are the boundary of 4LRV. Gray cell means the out of predictable range (negative value) in the models. Temperature *T* and log_10_ virus concentration *V* correspond to winter conditions. (*I*: initial ozone concentration, *k*: decay constant, *t*: contact time, *C*: Ct-value, *A*: assays to measure virus concentration, *W*: water types, *p*: pH, *T*: temperature, *V*: initial virus concentration).

**Fig. 7:**
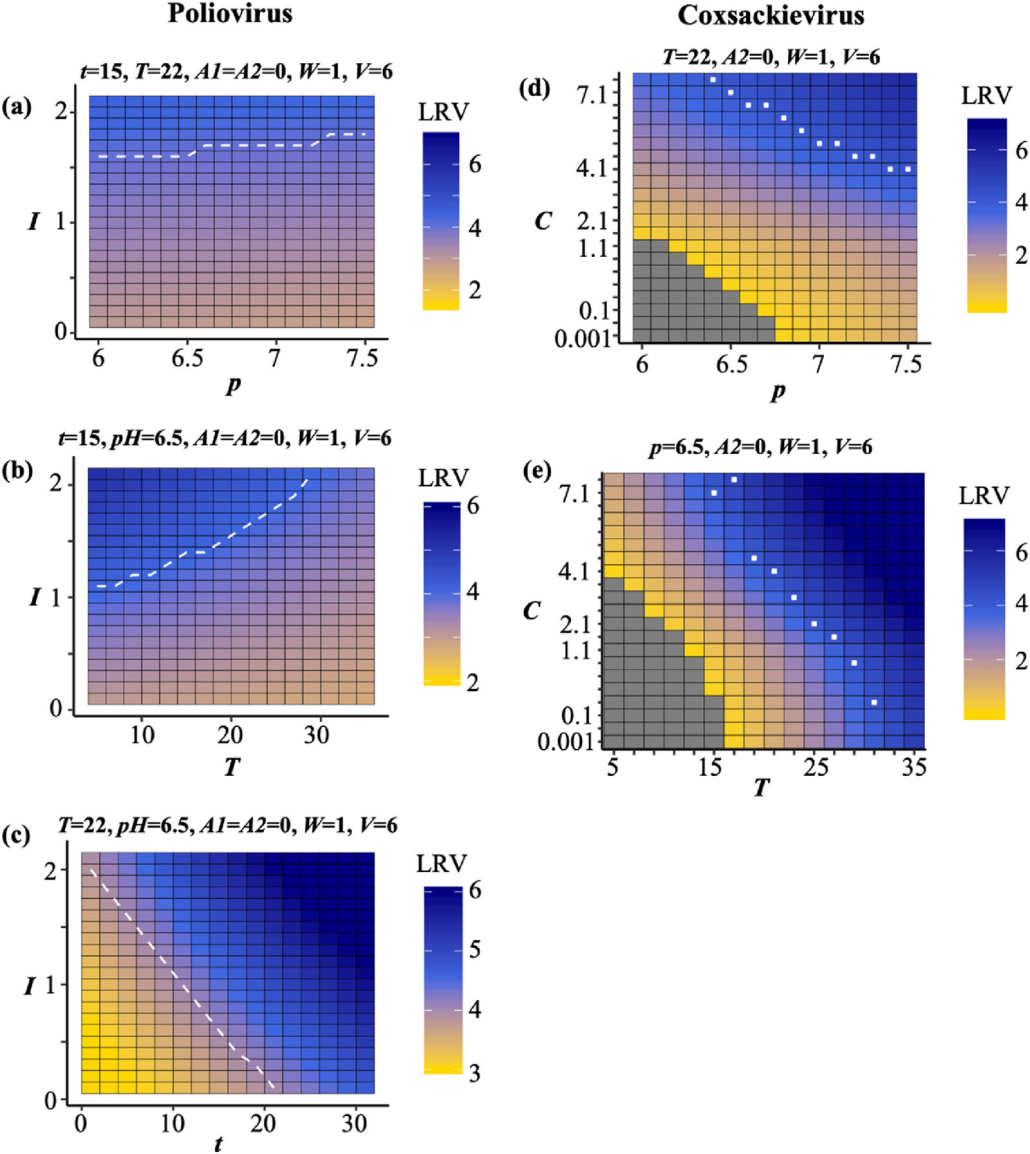
**Simulation of poliovirus and coxsackievirus LRVs**. Heatmaps indicate the LRVs by changing specific explanatory variables, and the white dotted lines are the boundary of 4LRV. The gray cell means the out-of-predictable-range value (negative value) in the models. Temperature *T* and log_10_ virus concentration *V* correspond to summer conditions. (*I*: initial ozone concentration, *k*: decay constant, *t*: contact time, *C*: Ct-value, *A*: assays to measure virus concentration, *W*: water types, *p*: pH, *T*: temperature, *V*: initial virus concentration).

The LRVs were generated using the major variables indicated in Fig. 5 to identify the predictable ranges in the models constructed and the disinfection conditions that achieved 4LRV. For all simulations, we set fixed values based on a practical WWTP in Sendai City, Japan, according to the seasonal prevalence of the viruses (summer: poliovirus and coxsackievirus, winter: norovirus and rotavirus). The *p* slightly affected the 4LRV achievement (Fig. 6a), but as the *k* increased, the 4LRV was achieved easily (Fig. 6b), which does not seem to be intuitive since a high value of *k* reduced the effectiveness of ozone disinfection. The rotavirus model clearly demonstrated the out-of-predictable ranges in certain *I* values (Fig. 6c, d). As well as norovirus, the *p* had a small effect on the LRV in the poliovirus model (Fig. 7a), but the higher *T* made it difficult to achieve the 4LRV in a disinfection reactor (Fig. 7b). The linear relationship between *I* and *t* was likely to be found (Fig. 7c). The coxsackievirus model also indicated unpredictable regions, and higher *p* and *T* made it easy to achieve 4LRV (Fig. 7d, e).

## 4 Discussion

In this study, we constructed ozone inactivation models predicting virus LRV by regularized regression analyses. The best performances for predicting norovirus and rotavirus LRVs were found in ARD with interaction terms, in which overfitting to training datasets could be avoided (Fig. 2, Fig. 3, Fig. 4 and Table 3). Bayesian ridge with interaction terms and lasso with quadratic terms indicated the best prediction performance for poliovirus and coxsackievirus, respectively, and had the ability to predict new datasets not relevant to the training dataset (Fig. 2, Fig. 3, Fig. 4 and Table 3). However, some test and training datasets have still not been predicted well (Fig. 4), and the HBM did not improve the prediction performances (Table 2). We also provided information about the unpredictable range of some variables and the desirable disinfection condition for the achievement of 4LRV (Fig. 6, Fig. 7).

**Table 3:** The best predictive inactivation models.

	Algorithm	Polynomial terms
Norovirus	Automatic relevance determination	Interaction
Rotavirus	Automatic relevance determination	Interaction
Poliovirus	Bayesian ridge	Interaction
Coxsackievirus	Lasso	Quadratic

The conventional kinetics of disinfection for viruses, such as the Chick-Watson, Hom and efficiency factor Hom (EFH) models, use only disinfection parameters (disinfectant concentration, contact time and decay constant) as model parameters (Chick, 1908; Watson, 1908; Hom, 1972; Haas and Joffe, 1994). Boehm et al. established the predictive model for the natural decay of enteric viruses, in which several water quality parameters were used as explanatory variables, and found the larger effects of water quality on natural decay rates (Boehm et al., 2019). The disinfection intensity of ozone is also affected by water quality parameters as the decay rate of ozone increases at higher temperatures (Sohn et al., 2004). Classical models are likely to be inadequate to predict LRVs under varied water quality status, but predictive water virology provides more practical models for LRV prediction using disinfectants because some operational and water quality information can be incorporated into the model (Fig. 5).

The selection of explanatory variables is critical for model construction (Abdul-Wahab et al., 2005). In fact, sparse estimation, which is applied for the predictive inactivation models except for poliovirus, implies a mitigation of overfitting to training datasets (Fig. 3), although an addition of polynomial terms is required to improve prediction performances. Ozone is generally consumed by organic contaminants in water (Park et al., 2001), and the established models lack variables related to disinfectant-consuming substances (*e.g.,* DOC and SS) (Sohn et al., 2004; Du et al., 2020), so the polynomial terms might compensate for the missing but essential variables. However, in this study, only the small number of variables was available and thus an addition of polynomial terms does not always support the prediction, which might attribute to the lack of fit to some datasets. To predict LRVs that are not predicted well with current models (Fig. 4), other types of water quality information needs to be incorporated into our models. The collection of datasets that completely involve water quality information is required to improve the prediction performance, which may be achievable by establishing a widely accessible database that includes datasets provided from laboratories and WWTPs, such as Global Water Pathogen Project (GWPP; https://www.waterpathogens.org/). If water quality information is difficult to prepare, the Ct-value that is calculated using a decay constant can be a substitution of disinfectant consuming matters (Kadoya et al., 2019). Additionally, we are able to construct the robust regularized regression-based models that exclude outliers from an estimation of model coefficients by using other machine learning algorithms (*e.g.,* Random Sample Consensus, Theil-Sen Estimator) (Fischler and Bolles, 1981; Dang et al., 2008).

Predictive water virology helps us identify the water quality and operational conditions that are required for 4LRV, but the models established here have to be updated continuously because the out-of-predictable range has remained large (Fig. 6, Fig. 7). The LRVs for norovirus, rotavirus and poliovirus are relatively stable under near-neutral pH levels (Fig. 6, Fig. 7), which is consistent with previous reports (Lin et al., 2014; Meng et al., 1987; Ward and Ashley, 1979). On the other hand, coxsackievirus LRVs are positively proportional to the pH-value since coxsackievirus is easily inactivated by higher pH (McGeady et al., 1979). Although waterborne viruses are easily inactivated with higher temperatures (Moresco et al., 2016; Biziagos et al., 1988), the model implies that poliovirus is inactivated more easily with low temperatures, which may be attributable to a data bias (*e.g.,* small datasets using low temperature, in which higher LRVs are observed). Also, as shown in Fig. 6b, an unintuitive result that the LRV became high as decay rate *k* increased is likely to be explained by the data bias owing to the small number of low *k* values (Fig. 1). Thus, virus LRV datasets obtained under various water quality conditions need to be collected to eliminate the data bias and expand the predictable range of models.

The predictive inactivation models established here are mostly based on datasets obtained in bench-scale experiments, so the models need to be carefully applied to LRV prediction at real-scale plants. In practice, there can be uncertainty related to hydraulics. For example, heterogeneity of DOC concentration, which is caused by the degree of incomplete mixing, could affect the decay of ozone concentration and the Ct-value. Also, under the mixing effect at a WWTP, water quality parameters need to be expressed using probability distributions (Sin et al., 2009 and 2011). Again, the model limitation must be solved by preparing a high-quality database of virus LRV, in which water quality parameters at WWTP are deposited. Alternatively, the prediction performance is developed by making the regularized regression-based models with explanatory variables that are expressed using probability distributions.

Several descriptions about the CL determination at WWTPs are available in the literature, but we do not have a common and flexible approach for such a determination (Hallowell, 2014). The current models for the LRV prediction help us determine the CL at the disinfection step at any WWTPs. The accepted concentration of virus in effluent is determined so as to correspond to the reference values of tolerable infectious risk and disease burden (*e.g.*, DALY loss per person per year) (USEPA, 1990; WHO, 2017). When a probability distribution of virus concentration in influent is known, the accepted virus concentration in effluent derives the target LRV (Ito et al., 2017). The operational and water quality parameters at WWTPs are monitored, and then we can apply the predictive inactivation models established in this study to determine the CL, which retains the target LRV.

## 5 Conclusions

Under the concept of predictive water virology, we provided the ozone inactivation models for waterborne viruses, which enable operators at a WWTP and risk assessors to determine the CL at the disinfection reactor. The proposed models are useful for predicting LRVs that are affected by some water quality parameters within the predictable range, and to make the models predictable, we need to continue to collect water quality information with LRV data and then update the models.

## Author agreement

SK and DS designed the present study. SK performed this study and prepared this article. ON, HK and DS checked the results of the analyses and modified the draft version of the article. All authors approved the final article.

## Declaration of competing interest

The authors declare that they have no known competing financial interests or personal relationships that could have appeared to influence the work reported in this paper.
