# Supplementary material for: Predictive water virology using regularized regression analyses for projecting virus inactivation efficiency in ozone disinfection
Source: Water Res X. 2021 Feb 12;11:100093. doi: 10.1016/j.wroa.2021.100093 (PMC7903012; doi:10.1016/j.wroa.2021.100093)

**Predictive Water Virology Using Regularized Regression Analyses for Projecting Virus Inactivation Efficiency in Ozone Disinfection**

Syun-suke Kadoya^a^, Osamu Nishimura^a^, Hiroyuki Kato^b^, Daisuke Sano^a,c*^

^a^Department of Civil and Environmental Engineering, Graduate School of Engineering, Tohoku University, Aoba 6-6-06, Aramaki, Aoba-ku, Sendai, Miyagi 980-8579, Japan

^b^New Industry Creation Hatchery Center, Tohoku University, Sendai, Miyagi, Japan.

^c^Department of Frontier Sciences for Advanced Environment, Graduate School of Environmental Studies, Tohoku University, Aoba 6-6-06, Aramaki, Aoba-ku, Sendai, Miyagi 980-8579, Japan

*Corresponding author: Daisuke Sano, Ph.D., Graduate School of Environmental Studies, Tohoku University, Aoba 6-6-06, Aramaki, Aoba-ku, Sendai, Miyagi 980-8579, Japan

Email: daisuke.sano.e1@tohoku.ac.jp

Tell: +81-11-795-7481

**Table S1. Information about strain types**

|  | ***N-Strain*** | ***Strain types*** |
| --- | --- | --- |
| Norovirus | 4 | Murine norovirus  Feline calicivirus  Human GII  Human 8FIIa |
| Rotavirus | 2 | Simian rotavirus  Human rotavirus |
| Poliovirus | 4 | Brunhilde  LSc  Mahoney  Sabine  (Unknown) |
| Coxsackievirus | 2 | A9  B5 |

**Table S2. Akaike’s information criteria of probability distributions appropriate for virus log reduction values**

|  | ***Normal*** | ***Log Normal*** | ***Gamma*** | ***Weibull*** | ***Exponential*** |
| --- | --- | --- | --- | --- | --- |
| Norovirus | 237 | 230 | 212 | 211 | 211 |
| Rotavirus | 245 | 257 | 240 | 238 | 240 |
| Poliovirus | 537 | 537 | 489 | 486 | 488 |
| Coxsackievirus | 246 | 257 | 240 | 238 | 243 |

**Table S3.** Variance inflation factor (*I*: initial ozone concentration, *k*: decay constant, *t*: contact time, *C*: Ct-value, *p*: pH, *T*: temperature, *A1*: infectivity or genome copy, *A2*: plaque forming unit or most probable number, *W*: water types, *V*: initial virus concentration, *S*: strain types)

|  | Multicol-  linearity | *I* | *k* | *t* | *C* | *p* | *T* | *A1* | *A2* | *W* | *V* | *S* |
| --- | --- | --- | --- | --- | --- | --- | --- | --- | --- | --- | --- | --- |
| Norovirus | ○ | 16.1 | 3.6 | 8.4 | 27.5 | 1.6 | 1.2 | 1.4 | - | 3.9 | 6.3 | - |
|  | × | 2.1 | 3.5 | 2.2 | - | 1.6 | 1.2 | 1.4 | - | 3.9 | 3.5 | - |
| Rotavirus | ○ | 4.7 | - | 10.5 | - | 1.3 | 12.7 | - | - | 71.8 | 25.8 | 1.7 |
|  | × | 3.2 | - | 5.5 | - | 1.3 | 9.7 | - | - | - | 5.8 | 1.6 |
| Poliovirus | × | 1.1 |  | 1.1 |  | 1.0 | 3.6 | 1.5 | 1.3 | 1.2 | 3.6 | - |
| Coxsackie  virus | × | 3.5 | - | - | - | 5.3 | 4.3 | 1.9 | - | 2.0 | 1.4 | 1.9 |

**Figure S1. Comparison of observed data with predicted values by the hierarchical Bayesian models using variables of the best regularized regression analyses (norovirus, rotavirus and coxsackievirus) or of variance inflation factor (poliovirus).** Error bars are the 95% confidence intervals.


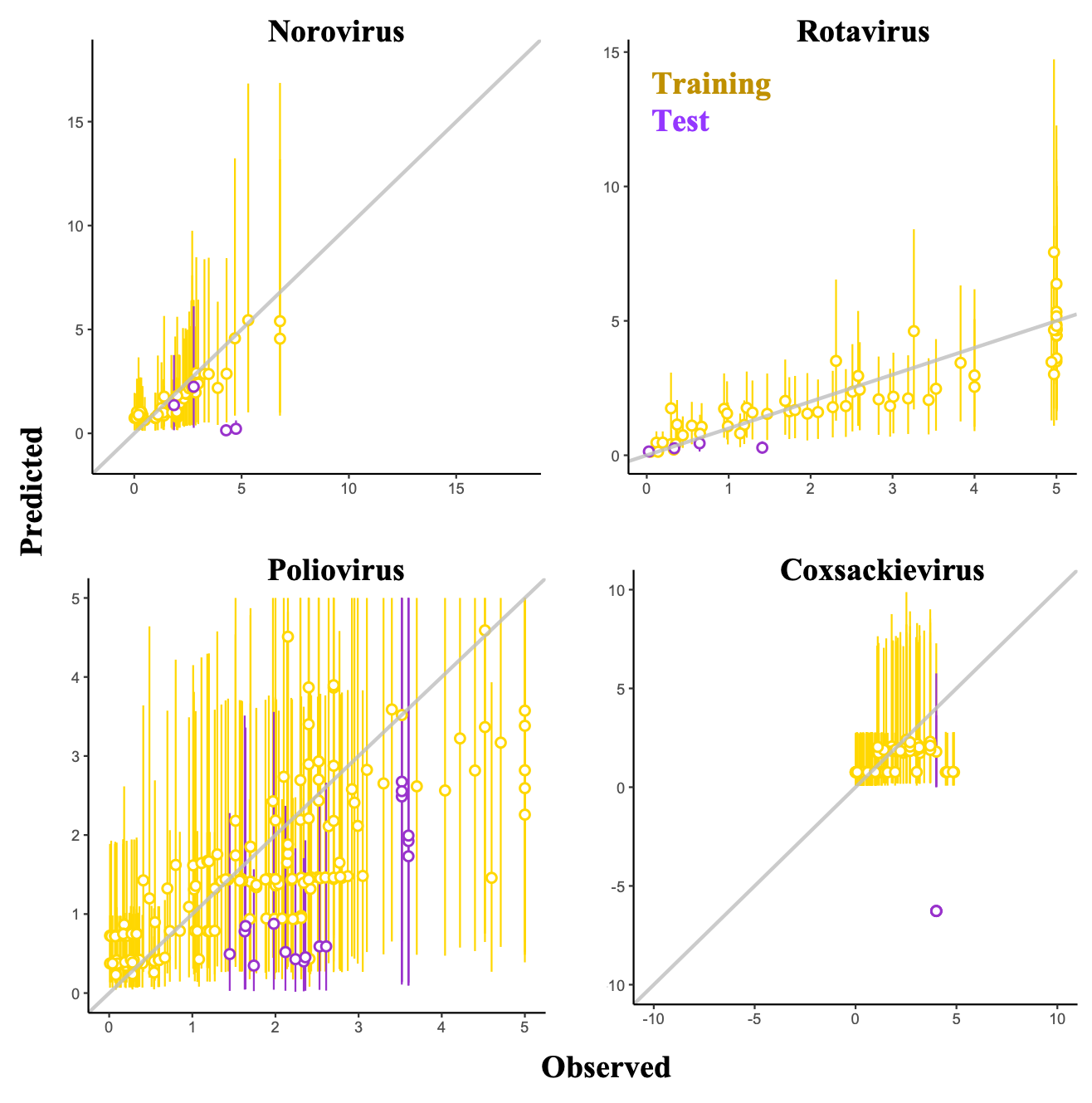


**Figure S2. Posterior distribution of the coefficients of hierarchical Bayesian models**


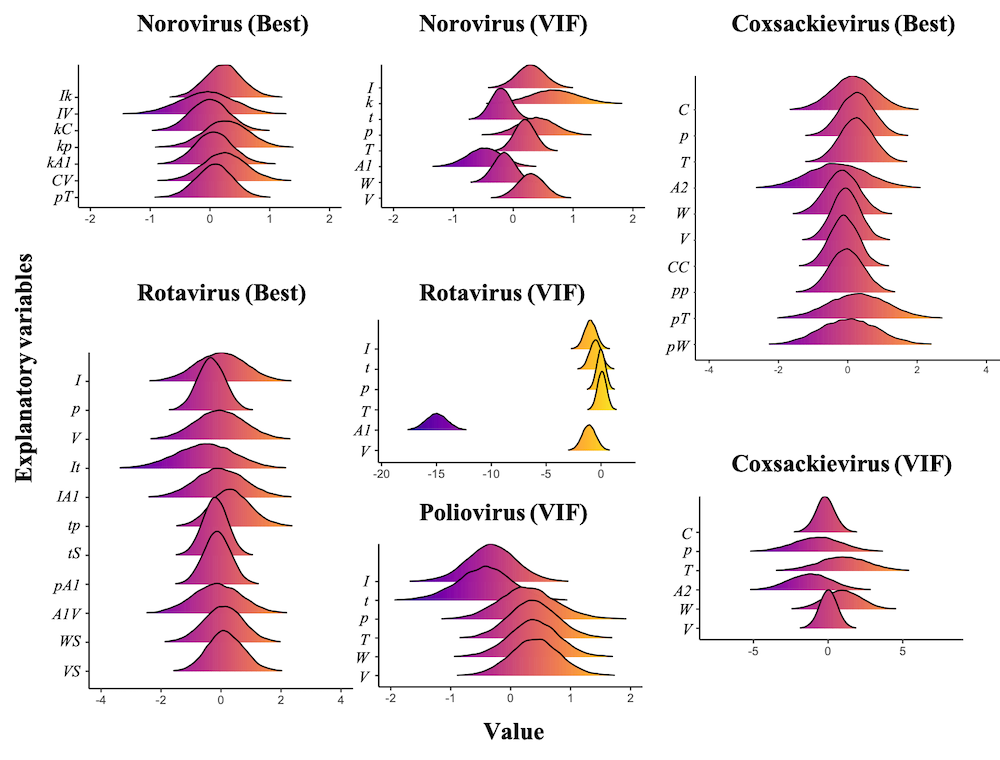

Supplement: Multimedia component 3 [file mmc3.docx]
